# Schisandrin B Antagonizes Cardiotoxicity Induced by Pirarubicin by Inhibiting Mitochondrial Permeability Transition Pore (mPTP) Opening and Decreasing Cardiomyocyte Apoptosis

**DOI:** 10.3389/fphar.2021.733805

**Published:** 2021-10-15

**Authors:** Hongwei Shi, Heng Tang, Wen Ai, Qingfu Zeng, Hong Yang, Fengqing Zhu, Yunjie Wei, Rui Feng, Li Wen, Peng Pu, Quan He

**Affiliations:** ^1^ Department of Radiation Oncology, Hubei Cancer Hospital, Tongji Medical College, Huazhong University of Science and Technology, Wuhan, China; ^2^ Department of Oncology, Renmin Hospital of Wuhan University, Wuhan, China; ^3^ Department of Cardiology, The First Affiliated Hospital of Chongqing Medical University, Chongqing, China; ^4^ Shenzhen Nanshan District People’s Hospital, Shenzhen, China; ^5^ Department of Vascular Surgery, The Second Affiliated Hospital of Nanchang University, Nanchang, China; ^6^ Department of Endocrine, The First Affiliated Hospital of Chongqing Medical University, Chongqing, China; ^7^ Department of Cardiology, Hubei Shiyan Taihe Hospital, Shiyan, China

**Keywords:** antiapoptotic, cardiotoxicity, schisandrin B, pirarubicin (THP), MPTP

## Abstract

**Objective:** Pirarubicin (THP), one of the anthracycline anticancer drugs, is widely used in the treatment of various cancers, but its cardiotoxicity cannot be ignored. Schisandrin B (SchB) has the ability to upregulate cellular antioxidant defense mechanism and promote mitochondrial function and antioxidant status. However, it has not been reported whether it can resist THP-induced cardiotoxicity. The aim of this study was to investigate the effect of SchB on THP cardiotoxicity and its mechanism.

**Methods:** The rat model of cardiotoxicity induced by THP was established, and SchB treatment was performed at the same time. The changes of ECG, cardiac coefficient, and echocardiogram were observed. The changes of myocardial tissue morphology were observed by H&E staining. Apoptosis was detected by TUNEL. The levels of LDH, BNP, CK-MB, cTnT, SOD, and MDA in serum were measured to observe the heart damage and oxidative stress state of rats. The expression of cleaved-caspase 9, pro/cleaved-caspase 3, Bcl-2/Bax, and cytosol and mitochondrial Cyt C and Bax was evaluated by western blot. H9c2 cardiomyocytes were cocultured with THP, SchB, and mPTP inhibitor CsA to detect the production of ROS and verify the above signaling pathways. The opening of mPTP and mitochondrial swelling were detected by mPTP kit and purified mitochondrial swelling kit.

**Results:** After 8 weeks, a series of cardiotoxicity manifestations were observed in THP rats. These adverse effects can be effectively alleviated by SchB treatment. Further studies showed that SchB had strong antioxidant and antiapoptotic abilities in THP cardiotoxicity.

**Conclusion:** SchB has an obvious protective effect on THP-induced cardiotoxicity. The mechanism may be closely related to the protection of mitochondrial function, inhibition of mPTP opening, and alleviation of oxidative stress and apoptosis of cardiomyocytes.

## Introduction

Pirarubicin (THP) is an anthracycline anticancer drug that has been widely used clinically. It has a broad antitumor spectrum and high clinical efficacy (Greish et al., 2005). However, it is also dose-dependent and exhibits cumulative cardiotoxicity (Von Hoff et al., 1979; Octavia et al., 2012). The clinical symptoms, such as arrhythmia and cardiac dysfunction, appear during the early treatment stages (Carvalho et al., 2014). Long-term anthracycline use has caused dose-dependent congestive heart failure and irreversible heart damage, which limits its clinical application (Casparini, 2006). Studies have shown that reactive oxygen species (ROS) and lipid peroxidation-induced oxidative stress and cardiomyocyte apoptosis play a leading role in THP-induced cardiotoxicity (Koh et al., 2002; Wang et al., 2017). Therefore, improving or alleviating oxidative stress injury and cardiomyocyte apoptosis might help to prevent and treat THP cardiotoxicity.

Schisandrin B has the ability to upregulate the cellular antioxidant defense mechanism and to promote mitochondrial function and antioxidant status, thus exhibiting a wide range of protective effects in many tissues in the body (Kim et al., 2004; Lam and Ko, 2012). Recent studies have found that long-term low-dose SchB treatment increases the function and antioxidant capacity of mitochondria in brain, heart, liver, and skeletal muscle of young and old rats (Chen and Ko, 2010; Lam and Ko, 2012). Based on the above results, we speculated that SchB might contribute to improving THP-induced cardiotoxicity. However, this hypothesis has not been confirmed *in vivo* or *in vitro*.

The present study investigated the antioxidant and antiapoptotic effects of SchB and explored their key pathways in THP-induced cardiotoxicity.

## Materials and Methods

### Materials

Pirarubicin and schisandrin B, purity ≥98%, were obtained from Shanghai Aladdin Reagent Co., Ltd. (Shanghai, China). Mitochondrial permeability transition pore (mPTP) inhibitor cyclosporin A (CsA) was purchased from MCE company. Brain natriuretic peptide (BNP), creatine kinase MB (CK-MB), and cardiac troponin T (cTnT) test kits were all purchased from Jiangsu enzyme label Biotechnology Co., Ltd. (Jiangsu, China). Malondialdehyde (MDA), superoxide dismutase (SOD), and lactate dehydrogenase (LDH) test kits were obtained from Nanjing Jian Cheng Biological Engineering Research Institute (Nanjing, China). The antibody of cleaved-caspase 9, pro/cleaved-caspase 3, Bcl-2/Bax, Cyt C, and COX IV were obtained from Cell Signaling Technology. All chemicals and reagents were of analytical grade.

### Animal Experiments

#### Animal Model

This study was performed according to the Guide for the Care and Use of Laboratory Animals and approved by the Animal Ethics Committee of the First Affiliated Hospital of Chongqing Medical University (CMU). 40 male SD rats (180–200 g) were obtained from the CMU experimental animal center. Rats were randomly distributed equally into four groups (n = 10 in each group): CON group (normal-diet-fed rats, equal volume of normal saline was injected into caudal vein), SchB group (SchB-diet-fed rats, 50 mg.kg^−1^, equal volume of normal saline was injected into caudal vein), THP group [normal-diet-fed rats; THP (3 mg.kg^−1^) was injected *via* caudal vein once a week], and THP + SchB group [SchB-diet-fed rats, 50 mg.kg^−1^; THP (3 mg.kg^−1^) was injected *via* caudal vein once a week]. Similar dietary feeding methods have also been published in our previous studies (Tang et al., 2021a; Tang et al., 2021b). The CON and THP group were fed with AIN-76A diet. AIN-76A diet contains approximately 5.2% fat (% by weight, approx. all from corn oil). SchB diet in the SchB group and SchB + THP group contains approximately 0.5‰ SchB in AIN-76A feed. After conversion, 0.5‰ SchB in diet = 50 mg/kg in rats. AIN-76A feed and the processing of SchB feed were performed by Jiangsu synergy pharmaceutical Bioengineering Co., Ltd.

The food consumption and body weight were measured twice a week.

#### Electrocardiogram and Doppler Echocardiography

The experiment ended at week 8. Rats were anesthetized with inhaled isoflurane (2%, maintenance dose was also 2%). Needle electrodes were inserted subcutaneously into the right upper limb, right lower limb, and left lower limb, respectively. The lead IV ECG was recorded by BL-420F biological function measurement system (Chengdu Taimeng Science and Technique Company). The R wave, S wave, T wave, and QT interval of rat ECG were measured on the system. The hair of the precordial region was removed, and the Doppler echocardiography was measured by Vivid E95 ultrasonic diagnostic apparatus with L8-18i-D probe (General Electric Company). When testing, the probe frequency was 18 MHz and the depth was 1.5 cm. EF, FS, LVIDd, and LVIDs in rat echocardiography were measured in the system. All operations were performed by professional ECG or echocardiography technicians, and the test results were checked by two or more cardiologists.

#### Sample Collection, Preparation, and Biochemical Indices

After fasting overnight, the rats were weighed and sacrificed under anesthesia. Blood samples were collected from abdominal aorta and centrifuged at 3,000 rpm for 30 min within 8 h. The supernatant was stored in a refrigerator at −80°C. The serum LDH, BNP, CK-MB, cTnT, SOD, and MDA were measured as soon as possible according to the operation procedure of the kit. Heart samples were excised and weighed. The remaining heart tissue was stored in the −80°C refrigerator.

#### Histopathological Examination

The left ventricular free wall myocardial tissue was fixed with 4% paraformaldehyde solution, embedded in paraffin, and cut into 5 μm thick sections. Then, HE staining and TUNEL staining were performed according to the kit instructions to observe the tissue changes. TUNEL apoptosis detection kit (green fluorescence) was purchased from Beyotime Biological Reagent Co., Ltd (Jiangsu, China). The paraffin section was dewaxed in xylene, dehydrated with absolute alcohol, washed with distilled water, and then added 20 μg/ml proteinase K without DNase (37°C for 30 min) and then washed with PBS for 3 times. 50 μL TUNEL solution was added to the target area and incubated at 37°C for 60 min. After washing with PBS for 3 times, the antifluorescence quenching sealing solution was used to seal the plates, which were observed with Nikon eclipse 80i microscope (Nikon, Chiyoda, Japan). Apoptosis level = apoptotic cells/total cells × 100%.

### Cell Experiments

#### CCK-8 Was Used to Determine the Optimal Concentration of SchB and the Coculture Effect of SchB and CsA

We used the CCK-8 cell activity test kit to explore the optimal concentration of SchB on cells and whether the coculture of SchB and CsA had additional effects. H9C2 cardiomyocytes were treated with 5 μM THP to establish the cell injury model. The concentration of SchB treatment was divided into 25 μM, 50 μM, 100 μM, and 200 μM to coculture with 5 μM THP to detect cell viability. 200 μM SchB was cultured alone to detect whether SchB had independent effects. In addition, the optimal concentration of SchB obtained was cocultured with 10 μm CsA to determine whether there was additional effect.

#### Cell Culture and Treatment

H9C2 cardiomyoblasts were purchased from the cell bank of Chinese Academy of Sciences and cultured in Dulbecco’s modified eagle’s high glucose medium (Gibco, China) and supplemented with 10% fetal bovine serum (Pan, Germany). The cell cultures were cultured in 5% CO_2_ incubator at 37°C. H9C2 cardiomyocytes were divided into six groups: normal group (CON), SchB group (SchB, 50 μm, 24 h), THP group (THP, 5 μm, 22 h), THP and SchB coculture group (SchB, 50 μm, 24 h + THP, 5 μm, 22 h), CsA group (CsA, 10 μm, 22 h), and THP and CsA coculture group (CsA, 10 μm, 24 h + THP, 5 μm, 22 h). In the SchB + THP and CsA + THP group, the cells were preincubated with SchB (50 μm) or CsA (10 μm) for 2 h and then cocultured with THP (5 μm) for 22 h.

#### ROS and Mitochondrial Permeability Transition Pore Staining in H9C2 Cells

H9C2 cardiomyocytes were seeded into 24-well plates and treated according to the cell treatment scheme mentioned above. When the cell growth reached 70–80%, the staining was carried out according to the instructions of ROS staining and mPTP staining kit. The fluorescent dye of ROS was DCFH-DA, and the fluorescent dye of mPTP was Calcein AM, which was observed with Nikon eclipse 80i microscope (Nikon, Chiyoda, Japan). The two fluorescent dyes were purchased from Beyotime Biological Reagent Co., Ltd. (Jiangsu, China). The positive area was counted by ImageJ software (ImageJ 1.51J8).

#### Swelling Experiment of Purified Mitochondria *In Vitro*

The cells were cultured according to the above cell grouping. When the cells grow to 70–80%, the mitochondria of each group of cells are extracted according to the instructions of cell mitochondrial extraction kit (Beyotime Institute of Biotechnology). Then, the swelling degree of mitochondria in each group was detected according to the instructions of the purified mitochondrial swelling photometric determination kit (Shanghai haling Biotechnology Co., Ltd.).

### Western Blotting

Mitochondrial and cytoplasmic proteins were extracted from cells and heart tissues according to the cell and tissue mitochondrial separation kit. The protein concentration in supernatant was determined by BCA kit. About 20–40 μg heart tissue or cell lysate was used for twelve alkyl sulfate polyacrylamide gel electrophoresis and then transferred to the FL membrane (microporous). The expression level of specific protein was standardized as GAPDH or COX IV.

### Statistical Analysis

Data were presented as mean ± SD. The significance of differences between groups was analyzed statistically using a one or two-way analysis of variance (ANOVA), followed by a Tukey’s multiple-comparison post hoc test. Differences were considered significant at *p* < 0.05.

## Results

### SchB Effectively Improved the THP-Induced Decrease of Body Weight and Food Intake in SD Rats

**FIGURE 1:**
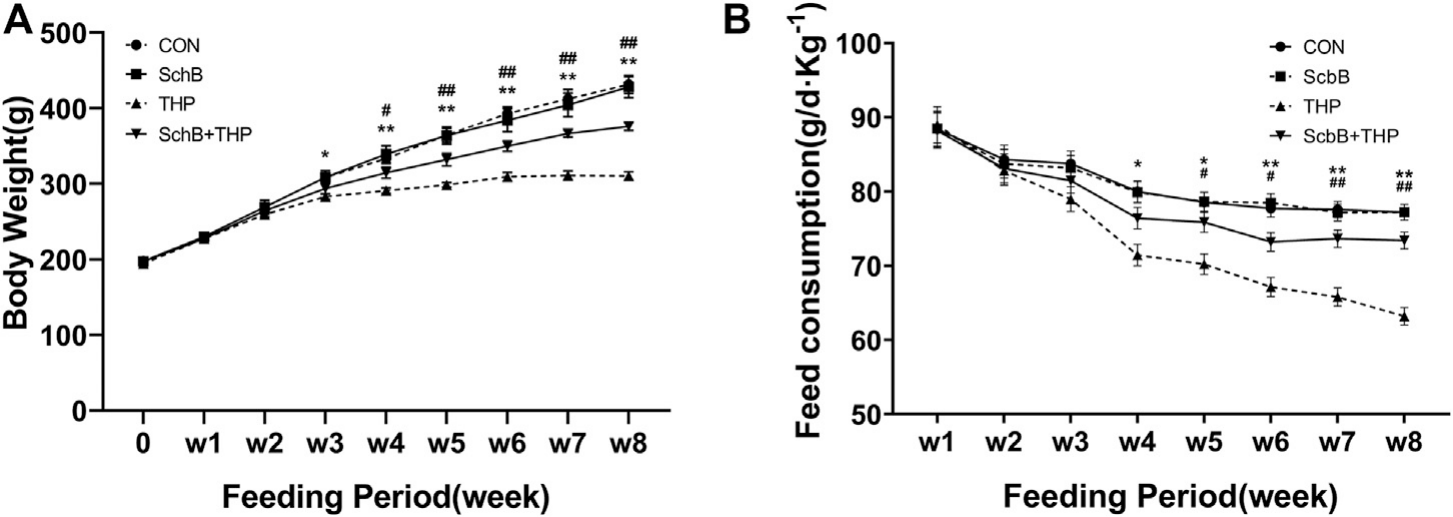
SchB effectively improved the abnormal body weight and food intake of SD rats induced by THP. **(A)** From the third week, the body weight of rats injected with THP alone was significantly lower than that of normal rats (THP vs. CON*, p < 0.05*). The weight loss of THP rats was further reduced after 4 weeks (*p < 0.01,* THP vs. CON). In THP rats treated with SchB (50 mg*kg^−1^), weight loss was significantly improved (*p < 0.05*, SchB + THP vs. THP). The improvement was more significant after the fifth week (*p < 0.01,* SchB + THP vs. THP). **(B)** From the fourth week, the food intake of rats injected with THP alone was significantly lower than that of normal rats (THP group vs. CON group, *p* < 0.05). The food intake of THP rats decreased further after 6 weeks (*p* < 0.01, THP and CON). In THP rats treated with SchB (50 mg*kg^−1^), the food intake increased significantly (*p < 0.05*, SchB + THP vs. THP). The improvement was more significant after the seventh week (*p < 0.01,* SchB + THP vs. THP). All values are mean ± SD. ^*^ *p < 0.05* vs. CON*,* ^**^ *p < 0.01* vs. CON; ^*#*^ *p < 0.05* vs. THP, and ^*##*^ *p < 0.01* vs. THP*.*

From the third and fourth week, the body weight (Figure 1A, *p* < 0.05) and food intake (Figure 1B, *p* < 0.05) of rats in the THP injection group decreased, and the difference was more significant at the fifth and sixth week (*p* < 0.01). After treatment with SchB, the above changes were significantly improved.

### SchB Effectively Improved the Changes of ECG and Echocardiography Induced by THP in Rats

**FIGURE 2:**
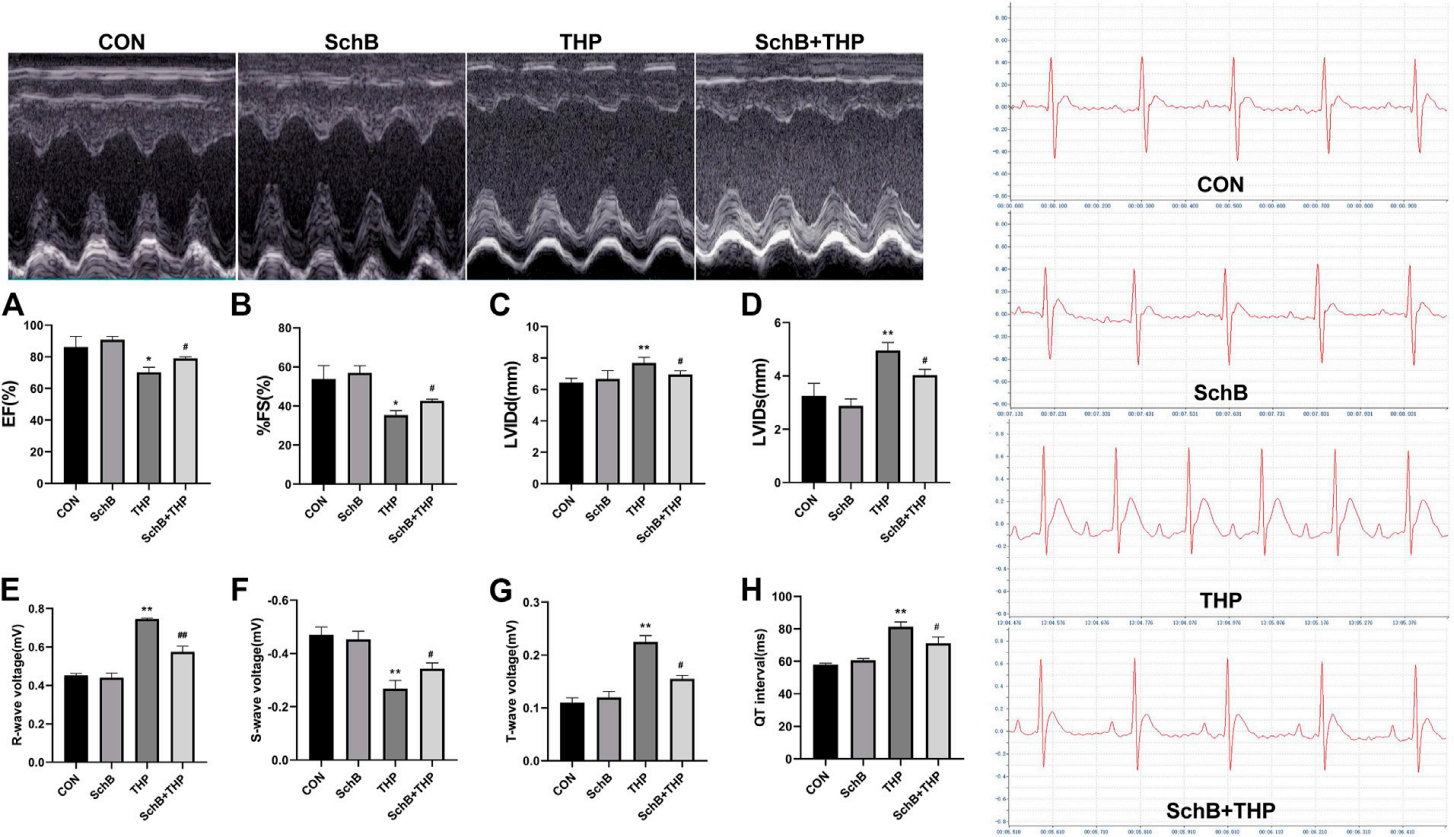
SchB effectively improved the echocardiographic and ECG changes of SD rats induced by THP. Compared with normal rats, EF **(A)** and % FS **(B)** decreased and LVIDd **(C)** and LVIDs **(D)** thickened (THP vs. CON). Compared with normal SD rats, the ECG of THP rats was also abnormal (THP vs CON). The R wave **(E)** and T wave **(G)** voltage increased; S wave voltage decreased **(F)**; and QT interval **(H)** prolonged (THP vs. CON). After treatment with SchB (50 mg*kg^−1^), the above changes were significantly improved (SchB + THP vs. THP). All values are mean ± SD. ^*^ *p < 0.05* vs. CON*,* ^**^ *p < 0.01* vs. CON*,* ^*#*^ *p < 0.05* vs. THP*,* and ^*##*^ *p < 0.01* vs. THP*.*

After 8 weeks, a series of ECG and echocardiogram (Figure 2) changes were observed in SD rats injected with THP alone, such as EF (Figure 2A) and FS (Figure 2B) decreased, LVIDd (Figure 2C) and LVIDs (Figure 2D) increased, R wave (Figure 2E) and T wave (Figure 2F) increased, S wave (Figure 2G) decreased, and QT interval (Figure 2H) prolonged. After SchB treatment, the above changes were significantly improved (Figures 2A–H, *p* > 0.05).

### SchB Improves Myocardial Tissue and Cardiac Index Changes Induced by THP in Rats

**FIGURE 3:**
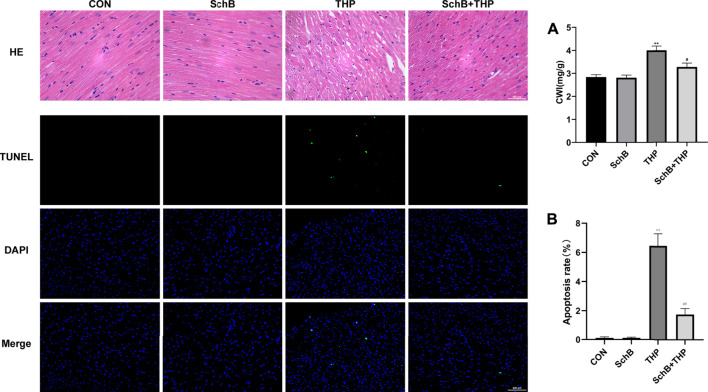
SchB attenuated THP-induced cardiac histopathological changes, apoptosis, and increased CWI. The myocardial tissue structure of the CON and SchB group was normal. In the THP group, the arrangement of cardiomyocytes was disordered, the intercellular space was significantly increased, and the cardiomyocytes were focal vacuolization or steatosis. The vacuolation and steatosis of myocardial cells in rats treated with SchB were significantly improved. TUNEL staining showed that there was no apoptosis in the control group and SchB group. In the THP group, there was partial apoptosis. In the group of SchB + THP, a few or a single cell apoptosis was occasionally observed. The CWI of rats in the THP group was significantly higher than that in the normal group, and the CWI of rats in the SchB + THP group was significantly lower than that in the THP group. All values are the mean ± SD. ^*^ *p < 0.05* vs. CON*,* ^**^ *p < 0.01* vs. CON*,* ^*#*^ *p < 0.05* vs. THP*,* and ^*##*^ *p < 0.01* vs. THP*.*

As shown by H&E staining in Figure 3, compared with the CON group, the arrangement of cardiomyocytes in the THP injection group was disordered, the intercellular space was enlarged, and the cardiomyocytes were focal vacuolization or steatosis. However, after treatment with SchB, the histological changes of heart were mild.

The cardiac weight index (CWI) of THP (Figure 3A) group was also higher than that of the CON group, and the above changes were relieved after SchB treatment.

TUNEL staining (Figure 3) showed that there was no cardiomyocyte apoptosis in the CON and SchB groups, but partial cardiomyocyte apoptosis in the THP injection group. Apoptosis of cardiomyocytes was occasionally observed after treatment with SchB. Quantitative analysis can be found in Figure 3B.

### SchB Alleviated the Changes of Blood Biochemical Indexes Induced by THP in Rats

**FIGURE 4:**
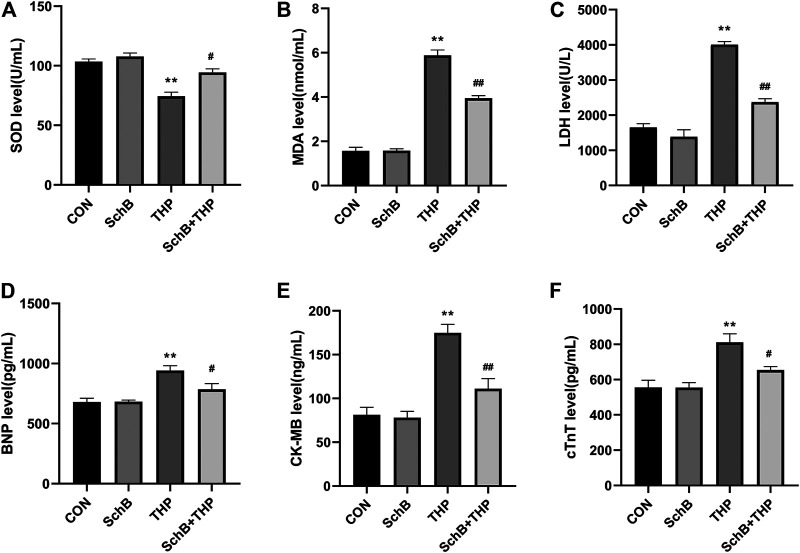
SchB effectively improved the level of serum markers in heart injury induced by THP: **(A)** superoxide dismutase (SOD) levels, **(B)** malondialdehyde (MDA) levels, **(C)** lactate dehydrogenase (LDH) levels, **(D)** brain natriuretic peptide (BNP) levels, **(E)** creatine kinase MB (CK-MB) levels, and **(F)** cardiac troponin T (cTnT) levels. All values are mean ± SD. ^*^ *p < 0.05* vs. CON*,* ^**^ *p < 0.01* vs. CON*,* ^*#*^ *p < 0.05* vs. THP*,* and ^*##*^ *p < 0.01* vs. THP*.*

In the blood, THP caused a decrease in SOD (Figure 4A) and an increase in MDA (Figure 4B), LDH (Figure 4C), CK-MB (Figure 4D), cTnT (Figure 4E), and BNP (Figure 4F). However, SchB treatment effectively improved these changes (Figures 4A–F, *p* > 0.05).

### The Effect of SchB and THP on the Expression of Apoptosis-Related Proteins *In Vivo*

**FIGURE 5:**
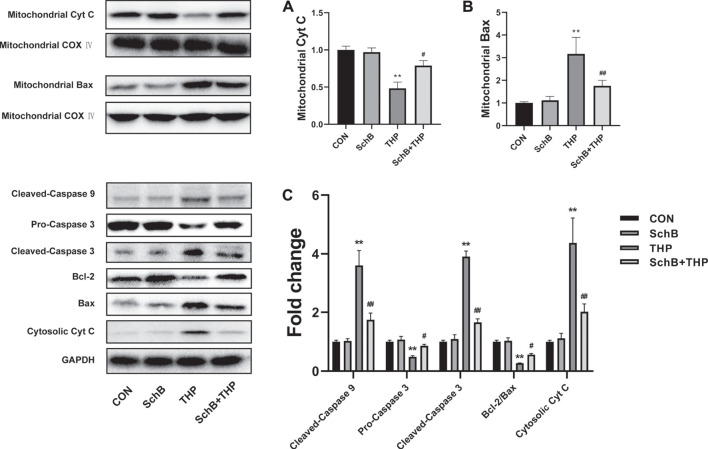
SchB ameliorates THP-induced abnormal expression of apoptosis-related proteins in rat heart. The content of mitochondrial Cyt C **(A)** decreased and Bax **(B)** increased in the THP group, but reversed in the SchB + THP group **(C).** In the THP group, the expression of cytosolic Cyt C and cleaved-caspase 9/3 increased, while procaspase 3 and Bcl-2/Bax ratio decreased. The above changes were reversed in the SchB + THP group. More evidence can be found in the semiquantitative analysis **(A–C)**. All values are mean ± SD. ^*^ *p < 0.05* vs. CON*,* ^**^ *p < 0.01* vs. CON*,* ^*#*^ *p < 0.05* vs. THP*,* and ^*##*^ *p < 0.01* vs. *THP*.

After 8 weeks of THP injection, the content of Cyt C (Figure 5A) decreased and Bax (Figure 5B) increased in mitochondria. In addition, the expression of procaspase 3 decreased, the expression of cleaved-caspase 3/9 and Cyt C increased, and the ratio of Bcl-2/Bax decreased (Figure 5C) in cytoplasm.

SchB treatment effectively alleviated the above changes. More evidence can be found in the semiquantitative analysis (Figures 5A–C).

### The Optimum Concentration of SchB and the Coculture Effect of SchB and CsA

**FIGURE 6:**
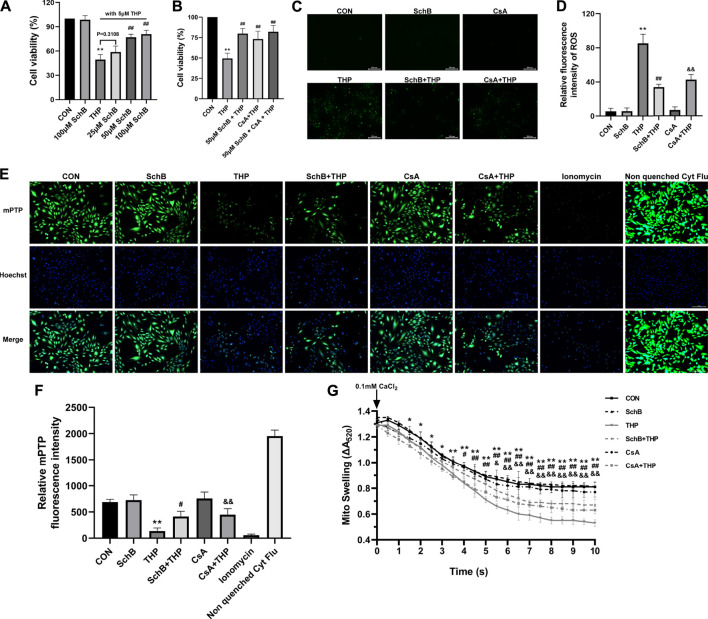
SchB ameliorates THP-induced abnormal expression of apoptosis-related proteins in rat heart. **(A)** Screening of optimum treatment concentration of SchB. **(B)** Observation on the effect of coculture of SchB and CsA. **(C)** ROS staining in each group. **(D)** Quantification of relative fluorescence intensity of ROS in each group. **(E)** H9C2 cells in CON, SchB, and CsA groups showed bright green fluorescence, while only weak green fluorescence was observed in the THP group. The green fluorescence was more obvious in SchB + THP and CsA + THP groups, which was greatly improved compared with THP. Ionomycin was a strong positive control. Nonquenched Cyt Flu was that the cytoplasmic fluorescence is not quenched. **(F)** Fluorescence quantitative analysis. **(G)** Mitochondrial swelling was analyzed for 10 min. All values are mean ± SD. ^*^ *p < 0.05* vs. CON*,* ^**^ *p < 0.01* vs. CON*,* ^*#*^ *p < 0.05* vs. THP*,* and ^*##*^ *p < 0.01* vs *THP.*

As shown in Figure 6A, under the condition of THP-induced cell injury, we detected the effects of four concentrations of SchB on cell activity. The results showed that THP significantly decreased cell activity. Although the cell activity of 25 μM SchB-treated cells increased slightly, there was no difference compared with the THP group. Three concentrations (50, 75, and 100 μM) of SchB treatment of cardiomyocytes significantly improved the decreased cell activity, but there is no difference among the three groups. The cells treated with the maximum concentration of 100 μM SchB also did not see significant cytotoxicity. Therefore, SchB concentration of 50 μM was selected for subsequent cell experiments.

The results of cotreatment of SchB and CsA showed that there was no significant difference in THP-induced cytotoxicity between SchB and CsA alone (Figure 6B).

### ROS Level of H9C2 Cardiomyocytes in Each Group

As shown in Figure 6C, THP induced H9C2 cells to produce a large amount of ROS, which decreased after both SchB and CsA treatment. ROS was almost absent in CON, SchB, and CsA groups.

### Effects of SchB, THP, and CsA on Mitochondrial Permeability Transition Pore in H9C2 Cardiomyocytes

As shown in Figure 6, H9C2 cells in CON, SchB, and CsA groups showed bright green fluorescence, while only weak green fluorescence was observed in the THP group. The green fluorescence was more obvious in SchB + THP and CsA + THP groups, which was greatly improved compared with THP. Ionomycin was a strong positive control.

### Effects of SchB, THP, and CsA on the Expression of Related Proteins in H9C2 Cardiomyocytes

**FIGURE 7:**
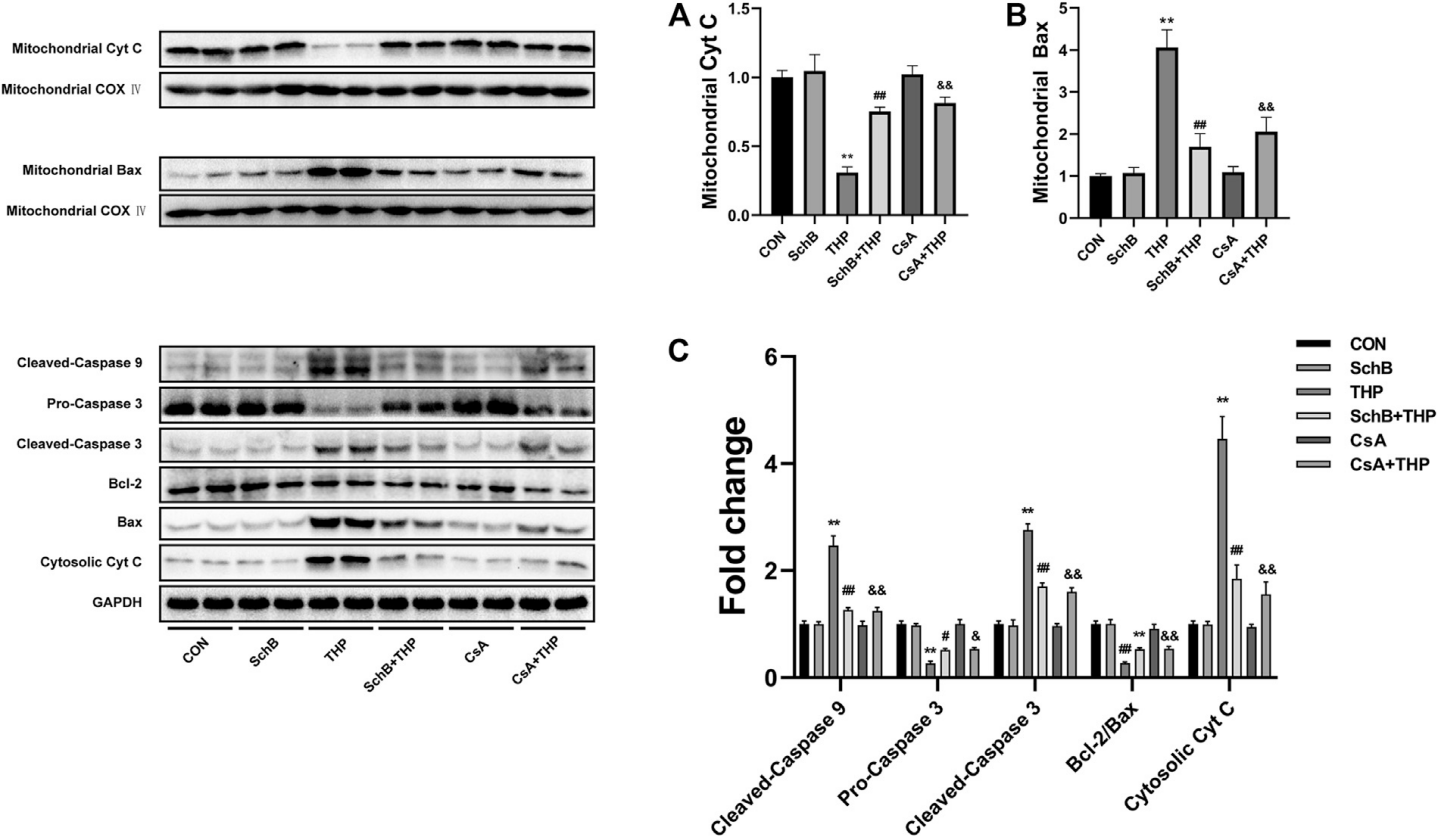
SchB ameliorates THP-induced abnormal expression of apoptosis-related proteins in rat heart. THP caused the decrease of Cyt C **(A)** and increase of Bax **(B)** content in mitochondria. The above changes were alleviated by the treatment of mPTP inhibitor CsA. SchB treatment showed similar effects. In addition, THP also increased the expression of Cyt C and cleaved-caspase 3/9, decreased the expression of procaspase 3, and decreased the ratio of Bcl-2/Bax in cytoplasm **(C)**. After treatment with SchB or CsA, the above changes were alleviated effectively **(C)**. More evidence can be found in the semiquantitative analysis **(A–C)**. All values are mean ± SD. ^*^ *p < 0.05* vs. CON*,* ^**^ *p < 0.01* vs. CON*,* ^*#*^ *p < 0.05* vs. THP*,* and ^*##*^ *p < 0.01* vs. THP*.*

As shown in Figure 7, THP caused the decrease of Cyt C content (Figure 7A) and the increase of Bax content (Figure 7B) in mitochondria. The above changes were alleviated by the treatment of mPTP inhibitor CsA (Figure 7). SchB treatment showed similar effect (Figure 7) in cytoplasm.

In addition, THP also increased the expression of cleaved-caspase 3/9 and Cyt C decreased the expression of procaspase 3 and decreased the ratio of Bcl-2/Bax (Figure 7C). After treatment with SchB or CsA, the above changes were alleviated effectively (Figure 7C). More evidence can be found in the semiquantitative analysis (Figures 5A–C).

## Discussion

Consistent with the expected results, THP caused cardiotoxicity in rats. After an intravenous injection of 10 mgkg^−1^ THP for 8 weeks, a series of systemic and cardiac toxicity changes occurred in SD rats, including abnormal body weight and food intake, adverse changes in echocardiography and electrocardiogram readings, cardiac tissue structure damage, abnormal increase in myocardial injury markers, oxidative stress injury, and increase in cardiomyocyte apoptosis. SchB effectively improved toxicity at a dose of 50 mgkg^−1^/D. Further studies have shown that the beneficial effects of SchB might be related to the inhibition of mPTP opening and decrease in cardiomyocyte apoptosis.

At present, it is generally believed that the mechanism of cardiotoxicity in anthracycline anticancer drugs involves oxidative stress, which increases cardiomyocyte apoptosis caused by ROS and calcium overload (Yao et al., 2015; Donato et al., 2017). High ROS level activates cytotoxic signals, leading to DNA damage, mitochondrial dysfunction, and decreasing protein synthesis. Consequently, this process induces cardiomyocyte apoptosis, leading to irreversible damage (Farías et al., 2017). Mitochondria are not only the main target of ROS damage but also an important site of ROS production (Donato et al., 2017). ROS oxidizes the thiol group of adenine nucleotide translocase (ANT) and causes the opening of mPTP (Li et al., 2018; Boyman et al., 2019). Presence of a large number of ROS results in apoptosis by destroying the mitochondrial membrane structure and releasing apoptosis-inducing factors (Zhang et al., 2019). At the same time, ROS also directly promotes the opening of mPTP, which leads to a decrease in mitochondrial membrane potential and the ROS bursting, forming a vicious circle and further aggravating mitochondria damage (Griffiths and Halestrap, 1995; Li et al., 2018). The mPTP is composed of nonspecific voltage-dependent anion channel (VDAC) located in the outer membrane, ANT located in the inner membrane, and Cyp D receptor located in the mitochondrial matrix (Fayaz et al., 2015). Under physiological conditions, only water and small molecular substances with a relative molecular weight of <1.5×10^3^ are allowed to pass through to maintain the electrochemical balance in mitochondria and form stable mitochondrial membrane potential (Mnatsakanyan et al., 2016; Green and Kroemer, 2004). Under various exogenous pathological stimuli, such as THP-induced heart injury, mPTP is opened abruptly, leading to mitochondrial membrane potential collapse, oxidative phosphorylation uncoupling, and ATP production disorder (Hausenloy and Yellon, 2003; Halestrap and Richardson, 2015). At the same time, the outer mitochondrial membrane ruptures, which promotes the release of Cyt C and other proapoptotic factors into the cytoplasm, initiate the caspase cascade apoptotic reaction and finally cause endogenous apoptosis. CsA is a classical mPTP inhibitor that specifically binds to Cyp D and inhibits the Cyp D and ANT binding, which also inhibits mPTP opening, reduces cell apoptosis, and plays a role in myocardial protection (Huang et al., 2014; Hurst et al., 2017). Studies have found that CsA pretreatment delays mPTP opening and improves cardiac systolic function and myocardial cell survival rate in cardiac surgery (Chiari et al., 2014).

Another interesting finding in this study was that SchB protected against THP-induced cardiotoxicity. Modern pharmacological studies have shown that SchB increases the activity or content of SOD and glutathione in tissue cells and resists the damage from free radicals to organisms (Ip and Ko, 1996; Xin et al., 2010; Hu et al., 2011; Jiang et al., 2015). Some scholars have found that MDA content in serum of MI/RI model rats treated with SchB was significantly lower than that of rats from the control group (Wang et al., 2003). In the present animal model, SchB increased SOD activity and decreased MDA content, which also fully reflected its strong antioxidant capacity. The latest research has shown that SchB significantly reduces the expression of p53 protein in a spinal cord injury mouse model and alleviates apoptosis in model mice (Xin et al., 2017). In addition, a study of ischemia-reperfusion injury in rats has found that the mitochondrial integrity of brain cells in the SchB treatment group was higher than that in the control group (Chiu et al., 2005). As mentioned above, mitochondria are the production sites of ATP and also the key organelle for oxidative stress and apoptosis. Mitochondrial dysfunction produces excessive active oxygen, which causes oxidative stress damage, opens up mPTP, and destroys ion homeostasis (Kim et al., 2003). Cyt C leaks into the cytoplasm, which leads to a series of events, such as caspase 9 activation and eventually mitochondrial-driven apoptosis (Jemmerson et al., 2005).

In general, SchB effectively inhibited the abnormal increase of oxidative stress induced by THP in rats. In addition, SchB seems to have the potential to inhibit mPTP opening similar to CsA and reduces the release of Cyt C from mitochondria to the cytoplasm. Finally, it alleviates cardiomyocyte apoptosis, which may be one of the molecular mechanisms of protecting myocardial mitochondria and preventing THP cardiotoxicity. However, the molecular mechanism of how SchB improves the increase of oxidative stress caused by THP *in vivo* is not clear. In addition, it is also not clear whether SchB directly affects mPTP opening like CsA. This is the limitation of our research and also the direction of our next work.

## Conclusion

Although THP is an anthracycline anticancer drug modified by adriamycin, its cardiotoxicity cannot be ignored. SchB has an outstanding potential in the prevention and treatment of THP cardiotoxicity in rats and myocardial cell injury models. Its mechanism may be related to the reduction in oxidative stress, inhibition of mPTP opening, and ultimate reversal of myocardial cell apoptosis. The present study suggested the possible mechanism of SchB in THP cardiotoxicity and provided a theoretical basis for the clinical application of SchB in the future.

## Data Availability

The original contributions presented in the study are included in the article/Supplementary Material. Further inquiries can be directed to the corresponding authors.
