# Corrigendum: Schisandrin B Antagonizes Cardiotoxicity Induced by Pirarubicin by Inhibiting Mitochondrial Permeability Transition Pore (mPTP) Opening and Decreasing Cardiomyocyte Apoptosis

**DOI:** 10.3389/fphar.2021.796551

**Published:** 2021-11-11

**Authors:** Hongwei Shi, Heng Tang, Wen Ai, Qingfu Zeng, Hong Yang, Fengqing Zhu, Yunjie Wei, Rui Feng, Li Wen, Peng Pu, Quan He

**Affiliations:** ^1^ Department of Radiation Oncology, Hubei Cancer Hospital, Tongji Medical College, Huazhong University of Science and Technology, Wuhan, China; ^2^ Department of Oncology, Renmin Hospital of Wuhan University, Wuhan, China; ^3^ Department of Cardiology, The First Affiliated Hospital of Chongqing Medical University, Chongqing, China; ^4^ Shenzhen Nanshan District People’s Hospital, Shenzhen, China; ^5^ Department of Vascular Surgery, The Second Affiliated Hospital of Nanchang University, Nanchang, China; ^6^ Department of Endocrine, The First Affiliated Hospital of Chongqing Medical University, Chongqing, China; ^7^ Department of Cardiology, Hubei Shiyan Taihe Hospital, Shiyan, China

**Keywords:** schisandrin B, cardiotoxicity, anti-apoptotic pirarubicin, anti-apoptotic, THP

In the original article, there was an error in the Funding section. We neglected to include “National Key R&D Program of China (Grant numbers: 2018YFC1311400, 2018YFC1311404)”.

The authors apologize for this error and state that this does not change the scientific conclusions of the article in any way. The original article has been updated.

